# Transmission electron microscope evidence of telocytes in canine dura mater

**DOI:** 10.1111/jcmm.12726

**Published:** 2015-12-29

**Authors:** Ting Xu, Shanshan Lu, Hongqi Zhang

**Affiliations:** ^1^ Department of Anatomy, Histology and EmbryologyShanghai Medical College of Fudan UniversityShanghaiChina; ^2^ Key Laboratory of Medical Imaging Computing and Computer Assisted Intervention of ShanghaiShanghaiChina

**Keywords:** telocytes, telopodes, transmission electron microscopy, dura mater

## Abstract

Telocytes (TCs) are a novel type of interstitial cells present in a wide variety of organs and tissues (www.telocytes.com). Telocytes are identified morphologically by a small cell body and specific long prolongations (telopodes) alternating thin segments (podomers) with dilations (podoms). The presence of TCs in rat meninges has been identified in previous research. We here present further evidence that TCs existed in canine dura mater, closed to capillary and surrounded by a great deal of collagen fibres under transmission electron microscope.

Telocyte (TC), a novel characteristic stromal cell, was firstly described in 2010 by Popescu's group as a case of serendipity 1. Telocytes were characterized with small cell bodies and telopodes (Tps), very thin and remarkably long prolongations. Telopodes formed a three‐dimensional network that may function as a scaffold to define the correct organization of tissues and organs 1, 2. In pathological condition, TCs displayed ultrastructural change, significantly reduced and progressively disappeared 3, 4. The presence of TCs has been described in a large variety of organs and tissues, such as oesophagus 5, eye 6, neuromuscular spindles 7, bone marrow 8, vasculature 9, cardiac valves 10, prostate 11, lung 12, liver 13, 14, oviduct 15, skin 16, salivary glands 17, uterus 18 *et al*. Telocytes present in rat meninges and choroid plexus were firstly reported by Popescu's group 19. The main objective of present research was to provide further credible evidence for the existence of TCs in canine dura mater under transmission electron microscope (TEM).

Three male beagles (23–26 kg, 3 months) were anaesthetized with diazepam (Shanghai Biochemical Pharmaceutical Company,?China) and ketamine hydrochloride (Fujian Gutian Pharmaceutical Company, ?China) by intramuscular injection. The anaesthetized animals were fixed in the supine position with their necks extended and thoracic cavities were opened, through a puncture in the left ventricle; 2500 ml of heparin physiological saline and 1000 ml 4% formaldehyde solution each animal were perfused at physiological pressure, respectively. After the perfusion, dura mater was removed. Transmission electron microscope was performed on small tissue fragments, processed according to routine Epon‐embedding procedure. And the processed specimen were observed under TEM (FEI TECAI SPIRITTEM, Eindhoven, The Netherlands), as previously described by Gherghiceanu and Popescu 20. Original TEM images were digitally processed by Adobe Photoshop CS5 to highlight TCs. All procedures were approved by the Ethic Committee for Animal Care and Use of Fudan University (The Ethics Committee approved number/ID: 20140226‐050), according to generally accepted international standards.

Telocytes with typical long and thin prolongations (Tps) were fundamentally identified in canine dura mater under TEM (Figs 1, 2, 3, 4). Frequently, TEM result revealed that TC cell bodies and Tps were usually surrounded by a great deal of collagen fibres (Figs 1, 2, 3, 4). The number of Tps was among 1–4 in 2D section, while the length of Tps ranged from 2.14 to 22.14 μm as we observed in dura mater (Figs 1, 2, 3, 4). Telopodes showed the features of moniliform aspect generated by the alternation of podoms (dilation of Tp) and podomers (slender segments; Fig. 1A). Telocytes with two thin and long Tps presented a fusiform shape (Fig. 1B). The TC showed typical features: a small oval body, and two processes which branch dichotomously (Fig. 2). Different TCs appeared to form networks by the interconnection of their Tps in dura mater (Fig. 3). Furthermore, the TC with long and slender processes was adjacent to the capillary (Fig. 4).

**Figure 1:**
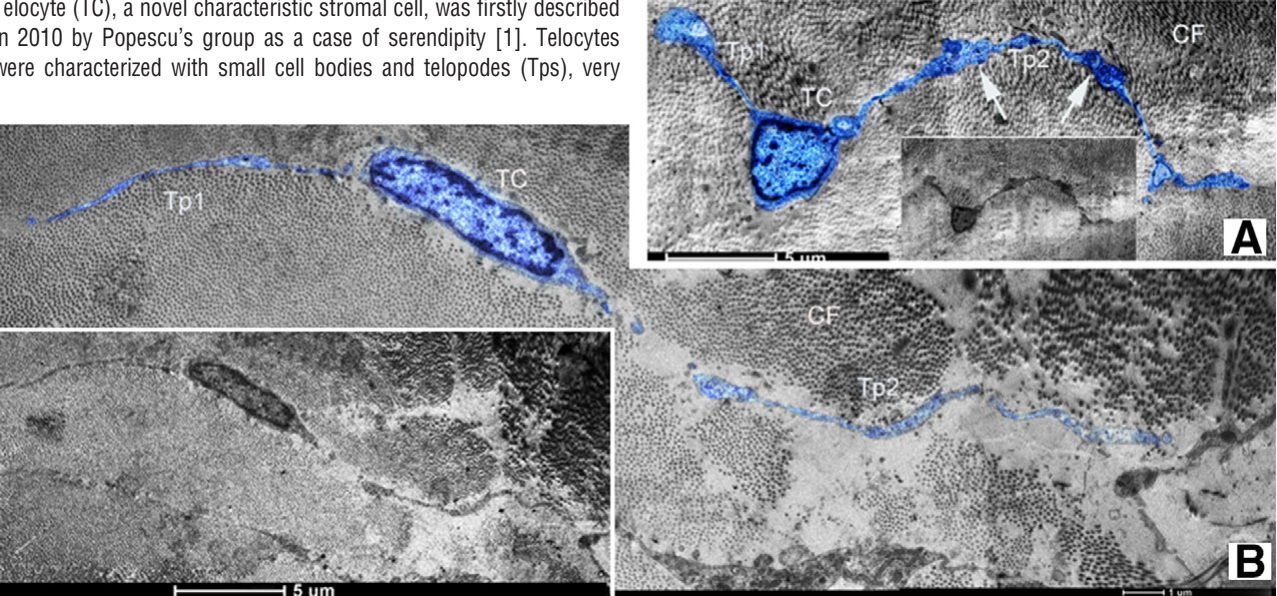
Transmission electron microscope (TEM) images of canine dura mater. (**A**) It shows a typical telocyte (TC) with two thin and long telopodes (Tps). The cell body of TC is round, which size is 6.56 μm in length and 1.44 μm in the average width. And the length of Tp1 and Tp2 is 8.34 μm and 15.11 μm, respectively. (**B**) Diameter of the TC oval cell body is 3.11 μm approximately. The morphological feature of the telopodes with thin segments (podomers) and dilations (podoms, white arrows) is shown. Two typical Tps are 4.67 μm (Tp1) and 15.33 μm (Tp2) in length; CF‐collagen fibre, bar =5 μm.

**Figure 2:**
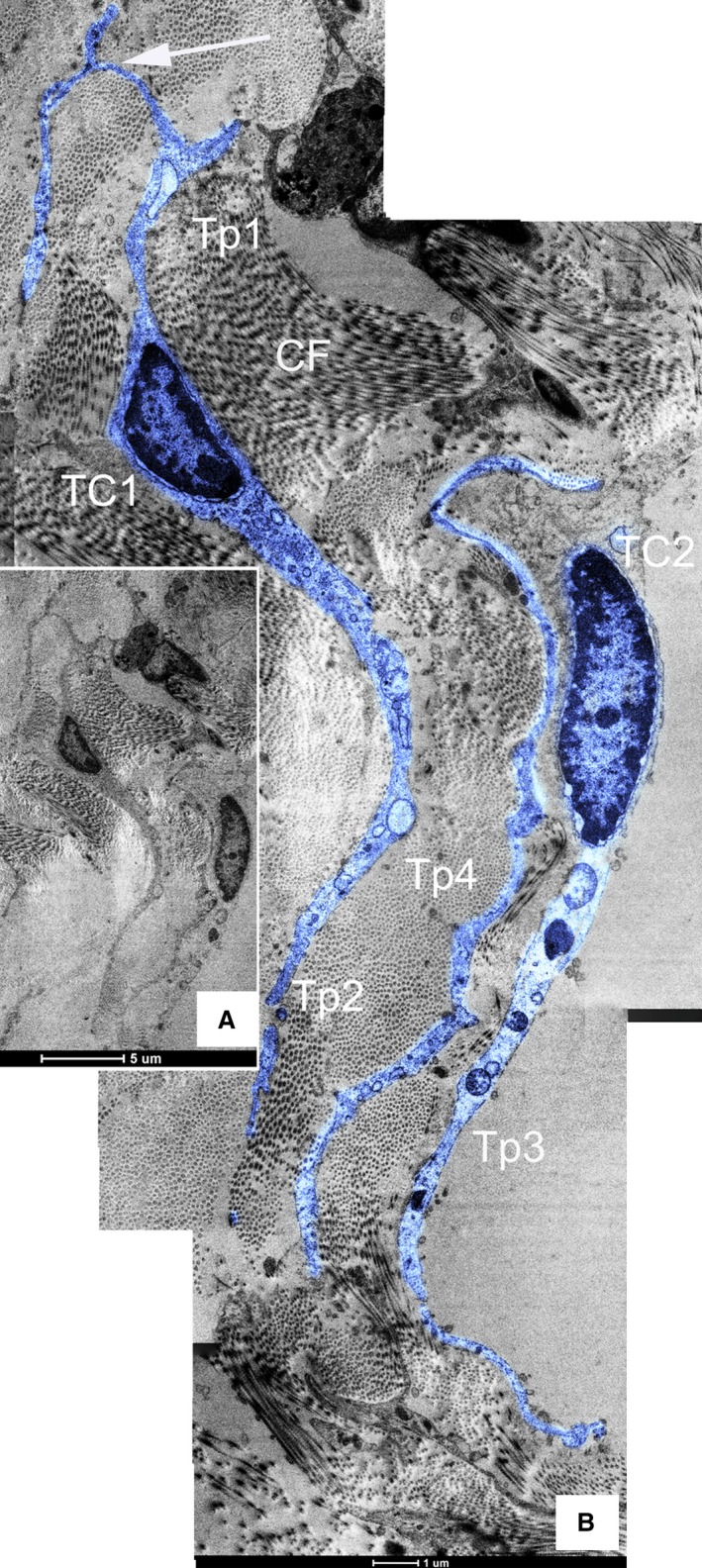
Transmission electron microscope (TEM) images of canine dura mater. (**A**) Two typical TCs are parallel with each other; bar = 5 μm. (**B**) It is the higher magnification of figure **A**. The body of TC1 takes on the shape of ellipse with 5.98 μm in length and the average width being 1.77 μm. The length of Tp1 and Tp2 are 11.07 μm and 13.31 μm, respectively. Tp1 presents a distinctive feature: bifurcation. The length of visible Tp3 of TC2 is 13.32 μm. Furthermore, a thin and long Tp4 whose cell body is not showed located between TC1 and TC2; CF‐collagen fibre; bar = 1 μm.

**Figure 3:**
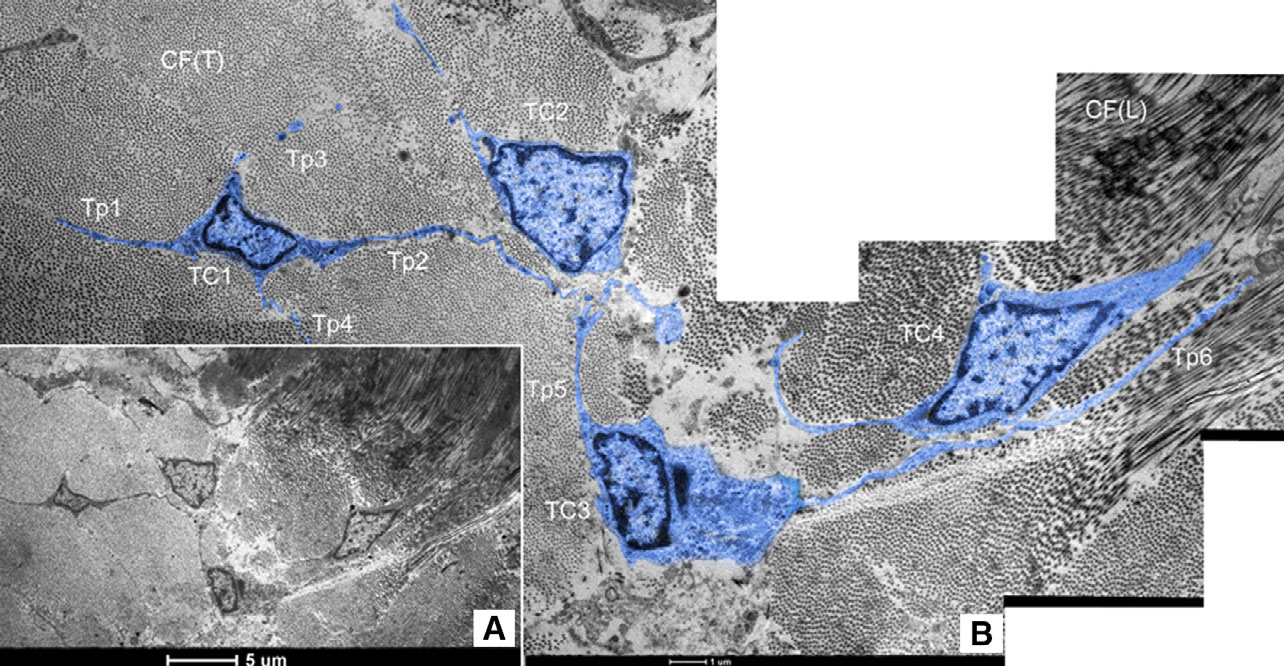
Cell communication between TCs in canine dura mater. (**A**) Four telocytes (named TC1–TC4 separately) form a network in dura mater; bar = 5 μm. (**B**) The higher magnification of **A** demonstrates the presence of end to end junction among telopodes (Tps). The cell body of TC1with four Tps (Tp1–Tp4) looks like quadrangular. Tp2, the longest process of TC1, reaches 7.14 μm forming close contact with TC2 and TC3. TC3 extends one shorter Tp5 (3.57 μm) and one longer Tp6 (22.14 μm); CF‐collagen fibre; CF(T)‐transverse section of collagen fibers; CF(L)‐longitudinal section of collagen fibres, bar = 1 μm.

**Figure 4:**
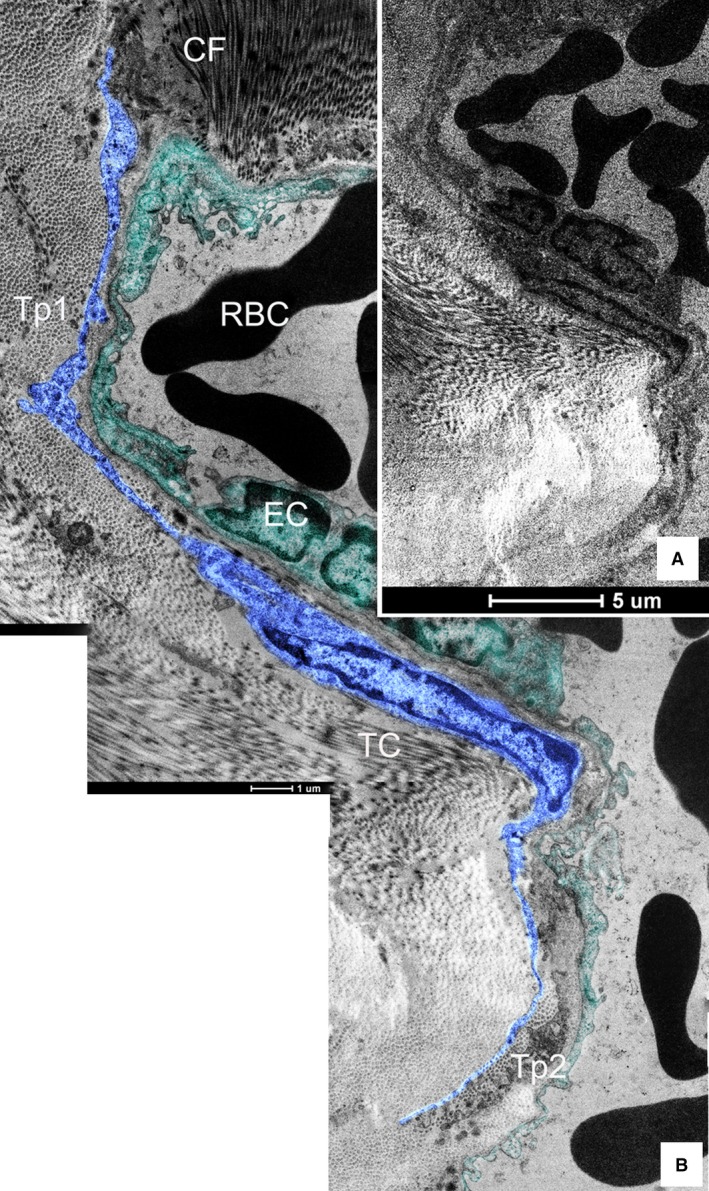
The topographic localization between the telocyte (TC) and the capillary of canine dura mater. (**A**) The strategic position of TCs, adjacent to capillary, is visible; bar = 5 μm. (**B**) It is the higher magnification of figure **A**. The cell body of one TC presents an irregular long fusiform with 8.89 μm in length and 1.97 μm in width. The Tps consisting of an alternation of podomers and podoms is 12.21/8.43 μm in length; CF‐ collagen fibre; RBC‐red blood cell; EC‐endothelial cell; bar = 1 μm.

The most distinct property of TCs is the existence of very long, thin and moniliform prolongations, termed Tps. So far, although no specific markers have been yet defined for TCs, the immunostaining with CD34/PDGFR‐alpha 21 or c‐kit 22 represents a useful marker for primary diagnosis of TCs. Under this condition, immunohistochemistry combined with TEM becomes the reliable method to precisely identify TCs. However, we failed to mark immunofluorescent labelling by reason of low density of TCs in canine dura mater. In the coming research, we will consider the matter of immunohistochemistry on dura mater TCs. Popescu and his colleagues 19 firstly reported the presence of TCs in rat meninges and choroid plexus. However, the meninges consist of three layers: the dura mater, the arachnoid mater, and the pia mater. Popescu's research did not detailedly indicate which layer TCs exist in the meninges. The aim of the present study was to explore whether TCs exist in dura mater. Our study indeed testified the existence of TCs in canine dura mater, based on the special appearance and the criteria for identification of TCs. Furthermore, TCs were usually surrounded by a great deal of collagen fibres forming a 3‐dimentional network through direct contact at junctions of Tps. Also, images of TEM showed that TCs were usually close to capillary. Dura mater, the outermost layer of meninges, play an important role in the protection of brain from injury 23. Previous studies also indicated TCs may have a significant role in assisting the differentiation and migration of stem cells during different neurogenesis stages 19. In addition, focuses on the latest progresses regarding TCs in the repair and regeneration of different tissues and organs including meninges and choroid plexus 19, 24, there are grounds for believing that TCs in dura mater might play apart in increasing the efficiency of collagen fibber and fibroblasts in the process of repair or regeneration. However, further studies are needed to explore the function of TCs in dura mater.

## Conflicts of interest

The authors confirm that there are no any conflicts of interest.
